# In Vitro Evaluation and Clinical Effects of a Regenerative Complex with Non-Cross-Linked Hyaluronic Acid and a High-Molecular-Weight Polynucleotide for Periorbital Treatment

**DOI:** 10.3390/polym17050638

**Published:** 2025-02-27

**Authors:** Hanadi Sami Abuyousif, Alexandre Porcello, Marco Cerrano, Cíntia Marques, Corinne Scaletta, Kelly Lourenço, Philippe Abdel-Sayed, Michèle Chemali, Wassim Raffoul, Nathalie Hirt-Burri, Lee Ann Applegate, Alexis E. Laurent

**Affiliations:** 1 For Ever Young Clinics, Jeddah 23431, Saudi Arabia; habuyousif@foreveryoungclinics.com; 2 Development Department, LOUNA REGENERATIVE SA, CH-1207 Geneva, Switzerland; a.porcello@louna-aesthetics.com (A.P.); c.marques@louna-aesthetics.com (C.M.); k.lourenco@louna-aesthetics.com (K.L.); 3 Aesthetic Surgery Department, Clinique Entourage, CH-1003 Lausanne, Switzerland; m.cerrano@entourage.ch; 4 Regenerative Therapy Unit, Lausanne University Hospital, University of Lausanne, CH-1066 Epalinges, Switzerland; corinne.scaletta@chuv.ch (C.S.); philippe.abdel-sayed@chuv.ch (P.A.-S.); nathalie.burri@chuv.ch (N.H.-B.); lee.laurent-applegate@chuv.ch (L.A.A.); 5 STI School of Engineering, Federal Polytechnic School of Lausanne, CH-1015 Lausanne, Switzerland; 6 Plastic and Aesthetic Surgery Service, Centre Médical Lausanne Ouest, CH-1008 Prilly, Switzerland; m.chemali@cmlo.ch; 7 Plastic and Reconstructive Surgery Service, Ensemble Hospitalier de la Côte, CH-1110 Morges, Switzerland; wassim.raffoul@ehc.vd.ch; 8 Center for Applied Biotechnology and Molecular Medicine, University of Zurich, CH-8057 Zurich, Switzerland; 9 Oxford OSCAR Suzhou Center, Oxford University, Suzhou 215123, China; 10 Manufacturing Department, LAM Biotechnologies SA, CH-1066 Epalinges, Switzerland; 11 Manufacturing Department, TEC-PHARMA SA, CH-1038 Bercher, Switzerland

**Keywords:** bio-stimulation, functional characterization, hyaluronic acid, hydrogel system, niacinamide, polynucleotide, skin collagen

## Abstract

Skin aging is a complex and multifactorial process influenced by both intrinsic and extrinsic factors. The periorbital area of the face is particularly susceptible to premature aging signs due to its delicate skin structure, and is a major concern for many individuals. While hyaluronic acid (HA)-based dermal filler products are commonly used for periorbital rejuvenation, novel approaches to effectively locally address the visible signs of aging are available. This study aimed to investigate Innovyal Regenerative Action (IRA), an injectable polynucleotide–HA (PN-HA) regenerative complex designed for periocular prejuvenation. Firstly, PN-HA was compared to other commercially available HA-based dermbooster products (Profhilo^®^, Suisselle Cellbooster^®^ Glow, and NCTF^®^ 135 HA) in terms of rheological properties, in vitro antioxidant capacity, and total collagen production stimulation in human fibroblasts. Secondly, the clinical effects of the IRA PN-HA complex were evaluated in two case reports (monotherapy for periorbital prejuvenation). It was shown that the PN-HA complex outperformed its comparators in terms of relative rheological behavior (biophysical attributes normalized to polymer contents), intrinsic antioxidant activity (CUPRAC, FRAP, and ORAC assays), as well as total collagen level induction (72-h in vitro dermal fibroblast induction model). Generally, the results of this study provided mechanistic and preliminary clinical insights into the potential benefits of the IRA PN-HA complex for periocular cutaneous treatment. Overall, it was underscored that combining the structural support and regenerative properties of PN with the hydrating and volumizing effects of HA bares tangible potential for multifactorial skin quality enhancement and for periocular prejuvenation in particular.

## 1. Introduction

Skin aging is a complex, progressive, and multifaceted process that is fundamentally influenced by a dynamic combination of intrinsic and extrinsic factors [1]. Of note, intrinsic aging is primarily governed and driven by genetic factors and the natural passage of time. This type of aging results in the gradual deterioration of both skin structure and function, which are characterized by thinning of the epidermis due to the progressive loss of collagen and elastin fibers within the dermis [1,2,3,4]. Extrinsic aging, on the other hand, is influenced by several environmental factors, such as ultraviolet (UV) radiation, pollution, smoking habits, and diet. Notably, exposure to UV radiation consistently leads to the in situ generation of reactive oxygen species (ROS) that damage cellular components, including DNA, proteins, and lipids [5,6,7,8].

Cosmetic treatment protocols classically leverage combination techniques and multiple products, such as topical therapies, skin resurfacing, dermal fillers, and other injectables [9]. Rapidly rising demands for effective preventive solutions are voiced by increasingly younger patients. The latter have pushed the evolution of prejuvenation treatments toward minimally invasive interventions (tweakments), enabling shorter downtimes and yielding immediate results [9,10,11]. In particular, younger patients increasingly focus on perioculars, which are very delicate and sensitive cutaneous regions around the eyes. These portions of the face are highly susceptible to fatigue and premature aging. Furthermore, this anatomical area is characterized by thinner skin compared to other parts of the face, making it more prone to developing fine lines, wrinkles, and sagging [12].

Currently, local injections of non-cross-linked (linear) hyaluronic acid (HA) are considered a gold standard and have demonstrated significant improvements in both skin hydration and elasticity. In the periorbital area of the face, they notably enable significant enhancements in skin brightness and texture [13,14]. Furthermore, the supplementation of HA hydrogels with biomolecules—including antioxidants, vitamins, and amino acids—is frequently employed to investigate potential additional enhancements or synergistic effects. This approach aims to optimize the biological outcomes of HA-based therapies by leveraging the complementary properties of the added compounds [14,15,16]. However, clinical information about the monotherapy usage of commonly applied facial cosmetic treatments, especially in the periorbital region, is not always available [17].

Recently, polynucleotide (PN)-based aesthetic product administration has gained widespread popularity among consumers and practitioners due to their reported clinical efficacy for skin rejuvenation [18]. Notably, a recent consensus report published by a team of Italian experts stated that PN fillers are safe and effective in treating the periocular area [19]. Of note, PN is a high-molecular-weight (MW) DNA polymer (i.e., ≥1500 kDa, as opposed to low-MW DNA polymers such as PDRN of <1500 kDa which are under the Marques Polynucleotide Cutoff) extracted from the gonads of salmon or trout [18]. PNs are terms that are specifically used to designate a polymer composed of several units of deoxyribonucleotides (including a phosphate group and a nitrogenous base). PNs have demonstrated promising effects in significantly promoting skin regeneration, reducing inflammation, improving skin texture, preventing scar formation, and mitigating wrinkles [18,19]. PN has been recently introduced by several reports describing this polymer as composed of 13 covalently linked nucleotide monomers with a high MW of up to 8000 kDa and a viscoelastic texture [19,20]. Due to its significant viscoelastic properties, PN forms a three-dimensional porous scaffold that provides lasting exogenous structural support in biological tissues. This property makes PN particularly effective for skin rejuvenation as it offers extended durability and a more robust framework for tissue repair [20,21]. Similar to PN, polydeoxyribonucleotide (PDRN) is composed of low-MW DNA fragments [19,20].

Upon the enzymatic breakdown of PN/PDRN, the activation of two main pathways (i.e., adenosine A_2A_ receptor binding and supply to the salvage pathway) stimulates the expression of vascular endothelial growth factor (VEGF), enhances fibroblast activity, promotes tissue repair, and deploys anti-inflammatory effects [22,23,24]. Importantly, the lower PDRN MW and the resulting lack of structural support make it less durable than PN for long-term tissue repair. Thus, PN is clinically used in cutaneous rejuvenation applications, aiming for an increase in dermal fibroblast populations, increased collagen production in the skin extracellular matrix (ECM), and improved tissue circulation. Additionally, PN locally increases fibronectin contents, which are essential for wound healing, tissue regeneration, and the maintenance of balanced isometric skin hydration [22,23,24,25].

As concerns such treatments, the literature shows the benefits of combining PN administration with non-insulated radiofrequency microneedling (RFMN) or with non-cross-linked HA fillers for periorbital rejuvenation and for reversing periorbital wrinkles [20,26,27]. Nevertheless, there is a gap in the literature when it comes to supporting the use of PN in monotherapy for skin prejuvenation and periocular treatments. Therefore, the aim of the present study was to perform in vitro functional characterization of an injectable PN-HA regenerative biopolymer complex (i.e., IRA, Innovyal Regenerative Action) for cutaneous prejuvenation, and preliminarily assess the clinical effects in case reports. The IRA PN-HA complex was compared with other commercially available dermbooster products (Profhilo^®^, Suisselle Cellbooster^®^ Glow, and NCTF^®^ 135 HA) containing non-cross-linked high-MW HA and additives in terms of rheology, antioxidant activity, and total collagen level stimulation potential. A simple and comparative method—namely standardizing the concentration of HA and total biopolymers—was implemented to assess the effects of the manufacturing processes on product viscosity and HA chain length. The commercial product exhibiting the closest mechanical properties to PN-HA (i.e., Profhilo^®^) was selected as a comparator for antioxidant capacity testing and collagen production stimulation assays using primary human dermal fibroblasts. Finally, we report the pilot clinical efficacy of the IRA PN-HA biopolymer complex for monotherapy of periocular cutaneous prejuvenation. Generally, the described protocol constitutes a significant step toward optimally addressing current patient preferences and needs in the context of periocular cosmetic interventions.

## 2. Materials and Methods

### 2.1. Reagents and Consumables Used for This Study

The Innovyal Regenerative Action^®^ (IRA, also referred to further as “the HA-PN complex”) product was obtained from Louna Aesthetics (Poisy, France). Based on manufacturer-supplied information, the PN-HA concentration in IRA is 12.5 mg/mL, comprising injectable-grade HA with a MW of 1.8 MDa at a concentration of 5 mg/mL, and injectable-grade PN with a MW of 1.5 MDa at a concentration of 7.5 mg/mL. These two polymers are combined by mixing with niacinamide to achieve physiological pH and osmolality. The Profhilo^®^ product was purchased from IBSA (Collina d’Oro, Switzerland). The Suisselle Cellbooster^®^ Glow (SCG) product was purchased from Suisselle (Zug, Switzerland). The NCTF^®^ 135 HA (NCTF) product was purchased from Filorga Laboratories (Paris, France). Collagen assay kits (i.e., reference MAK322) were purchased from Sigma-Aldrich (St. Louis, MO, USA). Pharmaceutical-grade purified water and sterile phosphate-buffered saline (PBS) buffer solutions were both purchased from Bichsel (Unterseen, Switzerland). TrypLE™, DMEM, and FBS cell culture reagents were all purchased from Life Technologies (Thermo Fisher Scientific, Waltham, MA, USA). Cell culture surfaces and disposable plastics were purchased from Greiner (Frickenhausen, Germany). Penicillin–streptomycin was obtained from the CHUV pharmacy (Lausanne, Switzerland).

### 2.2. Hydrogel Rheological Characterization Method

The basic rheological attributes of the selected commercial products were experimentally determined in oscillatory rheology using an HR 10 rheometer (TA Instruments, Guyancourt, France) equipped with a 40-mm-diameter Peltier plate–plate measuring system. The same geometry was used for all samples. The acquired rheological values represented measurements obtained within the linear viscoelastic region (LVR) of the amplitude sweep. The amplitude was set at 3 N/m^2^. Additionally, a standard frequency of 1 Hz—used for comparing injectable aesthetic products administered subcutaneously or intradermally—was applied. All measurements were performed at 25 °C on volumes of 600 μL for the hydrogel samples (i.e., each commercial hydrogel product). A sample hood was used during the measurements in order to minimize sample evaporation. The experimental storage moduli (G′), loss moduli (G″), and complex viscosity (ɳ*) values of the samples were determined using three experimental replicates in all the assays.

### 2.3. Product Antioxidant Capacity Determination Methods

For thorough characterization of the antioxidant capacity of the studied products, three different assays were used. Each of these assays measured antioxidant capacity through a different mechanism and/or against different reactive species. Specifically, using a panel of assays provided a more comprehensive assessment of the antioxidant properties of the investigated products, enabling authors to draw more robust conclusions.

#### 2.3.1. Cupric Reducing Antioxidant Capacity Determination

The CUPRAC assay measures the ability of antioxidants to reduce cupric ions (Cu^2+^) to cuprous ions (Cu^+^) obtained in a neutral pH environment. The CUPRAC assay (cupric reducing antioxidant capacity) was performed following an adapted protocol. Briefly, 50 µL of either the assay standards or the sample solutions were mixed with 150 µL of a reaction mixture, which consisted of equal volumes of copper (II) chloride solution (10^−2^ mol/L), ammonium acetate buffer (pH 7), and ethanolic neocuproine solution (7.5 × 10^−3^ mol/L). The components were mixed in a 1:1:1 ratio. After an incubation period of 60 min, absorbance readings were acquired using a microplate reader (Biotek Synergy Mx; BioTek Instruments, Luzern, Switzerland) at a wavelength of 450 nm. All measurements were performed in triplicate. Trolox standard solutions were used to generate the calibration curve for quantification of the antioxidant capacity.

#### 2.3.2. Oxygen Radical Antioxidant Capacity Determination

The ORAC assay evaluates the ability of antioxidants to protect a target molecule from oxidation by peroxyl radicals. These radicals are generated by the decomposition of AAPH at 37 °C and react with a fluorescent probe (fluorescein) at pH 7.0, leading to the formation of a non-fluorescent product. The ORAC assay (oxygen radical antioxidant capacity) was performed following an adapted protocol. The assay was conducted in a black, flat-bottom 96-well microtitration plate. Initially, 50 µL of fluorescein solution (8.4 × 10^−8^ mol/L) was added to each well to establish baseline measurements. Subsequently, 50 µL of either standards or sample solutions were introduced, followed by a 3-min shaking period and a 15-min incubation at 37 °C to allow the reaction to proceed. Immediately after the incubation, 50 µL of AAPH (2,2-azobis [2-amidinopropane] dihydrochloride) solution (153 mmol/L) were added to each well. Fluorescence was promptly measured using a microplate reader (Biotek Synergy Mx; BioTek Instruments, Luzern, Switzerland) with excitation and emission wavelengths set at 485 nm and 515 nm, respectively, and a reading height of 6 mm. Measurements were performed in triplicate. The protective effect of the samples was illustrated by the area under the curve (AUC) of the fluorescence reduction curve compared to a blank without antioxidant addition. Thus, the stronger the antioxidant, the longer the fluorescence values remained high, resulting in a higher AUC. The AUC was calculated using the trapezoidal integration approach presented in Formula (1):

AUC = 0.5 × (2 ∑ Fi − F41 − C) × Δt(1)

where Fi represents the fluorescence intensity recorded at each time point; ∑ Fi is the sum of all fluorescence values from time points 4 to 41; F41 is the fluorescence intensity at the final recorded time point (41); C is a correction factor; and Δt represents the time interval between consecutive fluorescence measurements. To determine the antioxidant capacity, the Net AUC was calculated by subtracting the AUC of the control (blank sample without antioxidants) from the AUC of the test sample (i.e., Net AUC = AUC sample − AUC blank).

#### 2.3.3. Ferric Reducing Antioxidant Power Determination

The FRAP assay measures the ability of antioxidants to reduce ferric ions (Fe^3+^) to ferrous ions (Fe^2+^) in an acidic medium. The FRAP assay was conducted using an assay kit (Sigma Aldrich, St. Louis, MO, USA). In a clear, flat-bottom 96-well microtitration plate, 10 µL of either ferrous standards or sample were combined with 190 µL of reaction mix in each well. After 60 min of incubation at 37 °C, the absorbance values were determined using a microplate reader (Biotek Synergy Mx; BioTek instruments, Luzern, Switzerland) at a wavelength of 594 nm. Measurements were performed in triplicate and multiple readings were obtained each hour for three hours.

### 2.4. Hydrogel Biological Evaluation in an In Vitro Dermal Fibroblast Model

Human primary dermal fibroblasts (i.e., DECH-2jM-Fib cell type, harvested from juvenile skin surgical waste tissue) were expanded in cell culture flasks containing 10 mL of Dulbecco’s modified Eagle medium (DMEM), supplemented with 10% fetal bovine serum (FBS) and 1% antibiotic–antimycotic solution. The primary cell cultures were statically incubated at 37 °C in a humidified atmosphere under 5% CO_2_. The cell growth was monitored daily using an inverted microscope. Subcultures were then performed twice per week when a cellular confluency level of 80% was attained and observed. Therefore, the confluent fibroblasts were transferred to a 24-well culture plate (i.e., 10^5^ cells/well). After 24 h, the cell culture medium was replaced with a mix of hydrogel sample/culture medium at a 1:1 ratio.

To evaluate their in vitro potential cytotoxicity and total collagen level stimulation attributes, IRA, Profhilo^®^, and PBS were incubated separately in contact with the human primary fibroblasts for 72 h. Following sample incubation, the culture supernatant was removed and the cell-seeded wells were washed multiple times with PBS. The cells were then detached, harvested with TrypLE™, and hydrolyzed through cycles of freezing and thawing in order to measure the total collagen contents by fluorescence with the collagen assay kit. Total collagen quantifications were performed in a two-step fluorescence protocol. In the first step of the procedure, the total collagen contained in the sample was enzymatically digested into peptides. Subsequently, the N-terminal glycine-containing peptides reacted with the dye reagent to form a fluorescent complex. The fluorescence intensity of this product—which was measured at λ_ex_ = 375/λ_em_ = 465 nm on a Varioskan LUX (Thermo Fisher Scientific, Waltham, MA, USA)—was directly proportional to the collagen concentration in the sample.

Finally, endpoint viability/metabolic activity determination was performed after 72 h of sample incubation on the adherent human primary fibroblasts using the cell proliferation reagent WST-1, according to the specifications of the manufacturer. The assay was carried out four times, using four experimental replicates.

### 2.5. Clinical Evaluation of IRA Safety and Efficacy in Periocular Prejuvenation

For the clinical evaluation, the IRA product was injected intradermally (30 G needle) using a multiple microaliquot technique in the perioculars of two female patients, with 1.0 mL applied on each side of the face. The treatment was performed twice with a two-week interval. Clinical follow-up imaging was performed after one or two months. After the injections, *Arnica montana* cream was locally applied as needed, supplemented by 20 mg of systemic Reparil^®^ three times per day for five days. Of note, *Arnica montana* cream is commonly used to reduce bruising and swelling, while Reparil^®^ is used to address potential inflammation following injection procedures. Both of these post-treatment applications are standard practice in aesthetic procedures involving facial injections.

### 2.6. Statistical Analyses and Data Presentation

The experimental data were reported herein as mean values accompanied by the corresponding standard deviations, which were plotted as error bars in the graphs. For the statistical comparison of values from multi-group quantitative datasets, a one-way ANOVA test or a two-way ANOVA test was performed and was followed by Tukey’s post hoc multiple comparison test. A *p*-value < 0.05 was retained as the general base for statistical significance determination. Detailed levels of statistical significance may be found in the Results Section and in the Supplementary Tables. The statistical calculations and/or data presentation were performed using Microsoft Excel (Microsoft Corporation, Redmond, WA, USA), Microsoft PowerPoint, and GraphPad Prism v. 8.0.2 (GraphPad Software, San Diego, CA, USA).

## 3. Results and Discussion

### 3.1. Formulation Design Considerations and Rheology Characterization

Importantly, when developing a novel regenerative complex based on HA hydrogel technology with the ability to reduce facial fine lines, the design considerations are critically related to product composition. This includes the choice of raw materials and concentrations (i.e., HA source, other polymers, vitamins, amino acids, or additives), as well as the manufacturing process. Specifically, terminal sterilization plays a major role in determining the final product’s ability to reduce skin fine lines (i.e., by significantly lowering the system’s viscosity) [28,29].

Herein, three commercial products (i.e., Profhilo^®^, Suisselle Cellbooster^®^ Glow [SCG], and NCTF^®^ 135 HA [NCTF]) were compared with the investigated IRA PN-HA complex (i.e., commercially available as Innovyal Regenerative Action^®^ [IRA]). Of note, these comparator products were selected because they all contain linear HA at concentrations higher than 5 mg/mL and possess documented bio-stimulatory properties. From a packaging viewpoint, IRA, NCTF, and SCG are available in vials of 3 mL, 5 mL, and 3 mL, respectively, whereas Profhilo^®^ is packaged in a 2 mL syringe. The registered product indications for the various test items are as follows:

- IRA or “HA-PN complex” is a bio-regenerative product designed to boost, regenerate, and protect the skin, indicated for full-face and décolleté treatments, including pre-laser care. It targets fine lines, acne scars, and general skin regeneration. It smooths fine lines, restores elasticity, boosts collagen and elastin production, hydrates, and repairs damaged skin, promoting overall firmness, smoothness, and plumpness;
- NCTF is indicated for cutaneous revitalization and for intense hydration of tired or dull skin, the filling of superficial wrinkles, and the re-plumping of mature skin or skin that lacks firmness;
- SCG is indicated for injection in the epidermis or dermis, enhancing microcirculation, improving skin structure, and reducing dryness or hyperkeratosis. It treats photoaging and hyperpigmentation, including melasma and chloasma;
- Profhilo^®^ is indicated for tissue remodeling and for the improvement in skin laxity of the face, neck, and body (Table 1) [30,31,32,33].

**Figure 1:**
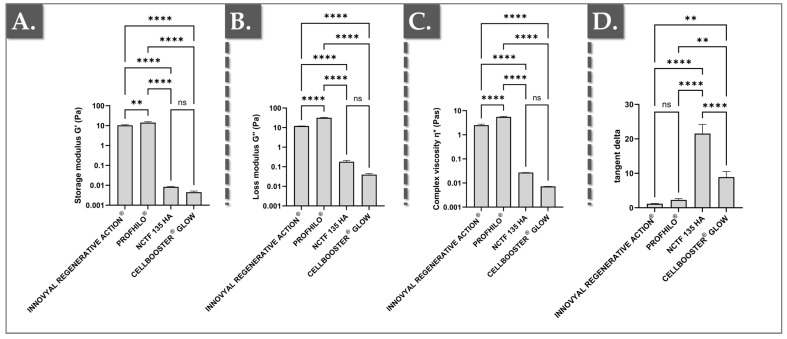
Rheological data for the investigated hydrogel systems. (**A**) Storage moduli (G′) comparison for the investigated products. (**B**) Loss moduli (G″) comparison for the investigated products. (**C**) Complex viscosity (ɳ*) comparison for the investigated products. (**D**) Tan δ value comparison for the investigated products. A significance level described by two asterisks “**” corresponds to a *p*-value between 0.001 and 0.01. A significance level described by four asterisks “****” corresponds to a *p*-value below 0.0001. Statistical analysis details are presented in Table S1. Numerical values for this dataset are presented in Table S2. ns, non-significant; Pa, Pascals; Pa·s, Pascal seconds.

The rheological analysis of the four injectable products (i.e., Innovyal Regenerative Action^®^, Profhilo^®^, NCTF^®^ 135 HA, and Suisselle Cellbooster^®^ Glow) revealed significant differences (Figure 1).

In detail, the complex viscosity ɳ* value of IRA was 2.51 ± 0.26 Pa·s, significantly higher than that of NCTF (i.e., approximately 10 times higher) and of SCG (i.e., approximately 350 times higher; Figure 1C). In contrast, Profhilo^®^ demonstrated the highest viscosity, approximately twice that of IRA (Figure 1C). Similarly, the storage modulus G′—indicating the material’s elastic properties—was much higher for IRA (i.e., 10.35 ± 0.72 Pa) and Profhilo^®^ (i.e., 14.17 ± 1.78 Pa), with values over 1000 times higher than those of NCTF and SCG (Figure 1A). Regarding the loss modulus G″, which measures viscous behavior, IRA and Profhilo^®^ showed significantly higher values (i.e., 12.00 Pa and 31.33 Pa, respectively) compared to NCTF and SCG, with a minimum difference of 60-fold (Figure 1B). Of note, the difference between IRA and Profhilo^®^ was more pronounced for G″ (i.e., Profhilo^®^’s G″ being 2.6 times higher), while the difference between IRA and the lower-moduli products (i.e., NCTF and SCG) was less marked compared to the differences observed in ɳ* and G′ values (Figure 1A–C).

The tangent delta (tan δ), or loss factor, was analyzed to gain deeper insights into the viscoelastic behavior of the four products. For reference, a tan δ value close to 0 indicates nearly ideal elastic behavior, while a value approaching infinity suggests nearly ideal viscous behavior. In this study, all of the investigated products exhibited tan δ values greater than 1, indicating a more pronounced viscous component. This finding is consistent with the literature as none of the tested products contain cross-linked HA, which would typically reduce the viscous component [34].

In detail, IRA and Profhilo^®^ demonstrated the lowest tan δ values (i.e., 1.16 ± 0.09 for IRA and 2.27 ± 0.37 for Profhilo^®^), reflecting a more balanced viscoelastic behavior with a slightly higher elastic component compared to the other products (Figure 1D). The lack of significant differences between these two products suggested that they possess similar viscoelastic properties, making them suitable for applications requiring a balance of elasticity and viscosity. In contrast, NCTF displayed the highest tan δ values (i.e., 21.55 ± 2.65), indicating a dominant viscous behavior, consistent with its role as a fluid with minimal structural support (Figure 1D). Finally, SCG showed intermediate tan δ values (i.e., 8.89 ± 1.65), indicating a significant viscous component though less extreme than that of NCTF (Figure 1D).

It should be noted that Profhilo^®^ stands out with its high HA concentration of 32 mg/mL and is the only product packaged in a syringe among those tested (Table 1). It presents the highest viscosity, G′, and G″ values, which is explained by its higher HA concentration among the tested commercial products (Figure 1, Table 1). Due to its elevated rheological properties, Profhilo^®^ can remodel different skin layers of the face and reduce wrinkles, in addition to its bio-stimulatory power, which enhances collagen and elastin production [32,33]. SCG, containing 6 mg/mL of HA along with vitamins and amino acids (i.e., processed via the CHAC technology), exhibited the lowest viscosity, G′, and G″ values among the tested products (Figure 1, Table 1). Designed to be injected into the epidermis or dermis to improve microcirculation, tropism, and to restore skin structure, its remodeling and fine line reduction capabilities are expected to be limited [31].

In comparison, both NCTF and IRA are dispensed in vials and contain 5 mg/mL of HA. Of note, NCTF is indicated for cutaneous revitalization, the hydration of tired or dull skin, and for the treatment of superficial wrinkles, while IRA is a bio-regenerator aimed at restoring the skin ECM, improving skin elasticity, and reducing the appearance of wrinkles [30]. Despite having the same HA concentration, IRA demonstrated significantly higher rheological values for viscosity, elastic modulus (G′), and viscous modulus (G″) compared to NCTF and SCG (Figure 1, Table S1). Of note, the rheological properties of IRA were closer to those of Profhilo^®^, despite having a similar HA concentration to NCTF and SCG and approximately 6 times less HA than Profhilo^®^ (Figure 1, Table 1).

**Figure 2:**
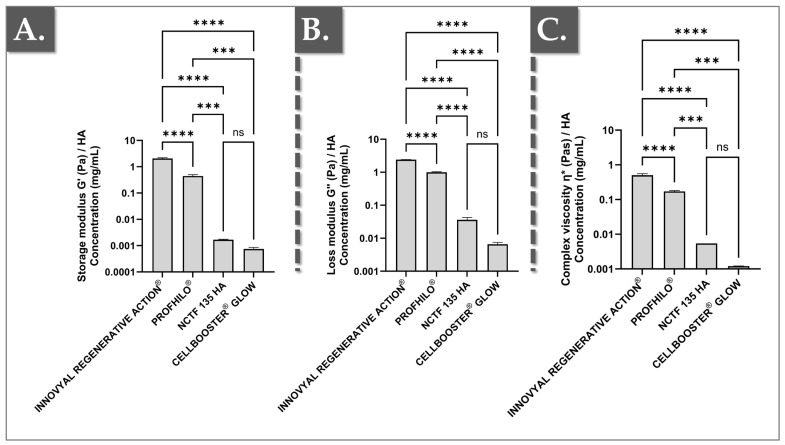
Rheological data analysis complementing the results presented in Figure 1. The rheological values were normalized to the respective total HA contents of the products. (**A**) Normalized storage moduli (G′) comparison for the investigated products. (**B**) Normalized loss moduli (G″) comparison for the investigated products. (**C**) Normalized complex viscosity (ɳ*) comparison for the investigated products. A significance level described by three asterisks “***” corresponds to a *p*-value between 0.0001 and 0.001. A significance level described by four asterisks “****” corresponds to a *p*-value below 0.0001. Statistical analysis details are presented in Table S3. HA, hyaluronic acid; ns, non-significant; Pa, Pascals; Pa·s, Pascal seconds.

Beyond polymer concentration, the MW of the HA chains plays a crucial role in product viscosity attributes, with longer chains typically resulting in higher viscosity [35,36]. However, during manufacturing, HA chains are subjected to various stresses, particularly during homogenization and sterilization processes. Additionally, the other compounds mixed with HA can influence viscosity (i.e., either positively or negatively) [36,37,38]. Based on this consideration, the strict technical benchmarking of HA-based hydrogel systems from different manufacturers would require biophysical attribute normalization to the polymer content. Therefore, the results presented in Figure 2 compare the ratios of complex viscosity, G′, and G″ to the respective HA concentrations across the different products.

In detail, given the significant influence of polymer concentration on product rheological parameters, the rheological values presented in Figure 1 were normalized based on the HA concentration of each product (Figure 2). Thus, by eliminating the HA concentration variable, these normalized ratios offer clearer insights into each formulation’s ability to maintain mechanical properties, independent of HA content. Additionally, this approach indirectly reflects the relative length of the HA chains present in the products (Figure 2).

Notably, IRA exhibited significantly higher ratios of complex viscosity, storage moduli, and loss moduli compared to the other three formulations. Interestingly, these normalized values were also significantly higher than those observed for Profhilo^®^ (Figure 2). Importantly, IRA uses HA with a MW of 1800 kDa, which partly explains the observed higher rheological values. In contrast, Profhilo^®^ employs the NAHYCO™ technology, combining high- and low-MW HA (i.e., 1100–1400 kDa and 80–100 kDa) [32,39]. The inclusion of PN (or sodium DNA) at 7.5 mg/mL in IRA further enhances its viscosity due to the high MW (i.e., 1800 kDa) of the raw material used in the Boost & Shield^®^ technology (Table 1).

Due to the combination of PN and HA in IRA, the formulation forms a regenerative biopolymer complex with a total biopolymer concentration of 12.5 mg/mL. To fully account for these factors (i.e., the combined influence of PN and HA on the viscosity attributes of the system), Figure S1 normalizes the product rheological parameters to the gross biopolymer concentration. Therein, even when accounting for the presence of PN, IRA maintained the highest ratios, with the G′ values reported as significantly higher than that of Profhilo^®^ (Figure S1).

The third explanation for the observed differences in the ratios presented in Figure 2 and Figure S1 relates to the product manufacturing process. Indeed, the degree of stress sustained by the system during mixing and hydration, especially during sterilization, can break HA chains, thereby greatly affecting viscosity [28,40,41]. Among the studied products, IRA, Profhilo^®^, and SCG undergo final sterilization by steam or heat, while NCTF is aseptically processed without terminal heat treatment. Surprisingly, despite the lack of terminal heat sterilization, NCTF exhibited significantly lower rheological values compared to IRA, potentially indicating the presence of very-low-MW HA chains in NCTF (Figure 1, Figure 2 and Figure S1).

The fourth explanation involves the other components/excipients in the comparator formulations. Indeed, Profhilo^®^ contains only HA, whereas IRA includes HA, PN, and niacinamide (vitamin B3; Table 1). Furthermore, SCG contains two vitamins and six amino acids, while NCTF has over fifty-five listed ingredients (Table 1) [42]. It should be stressed that additives can positively or negatively influence the mechanical properties of the finished product. In detail, the literature describes that some polyols tend to protect HA chains, whereas vitamin C or lysine—present in SCG and NCTF—may degrade HA chains [43]. Given the rheological properties of SCG and NCTF, which are close to those of water, and the complexity of their formulations (i.e., more than seven ingredients with limited information on concentrations), these products were excluded from further experimental analysis. Specifically, their direct comparison with IRA in terms of antioxidant and total collagen level stimulation potential was deemed inappropriate due to the significant differences in product formulation and mechanical properties.

Finally, with regards to the interactions between HA and PN, the authors assess that the combination of these two biopolymers procures most of the necessary and sufficient product rheological property modification effects. Since PN is a high-MW biopolymer, it impacts the rheology of the formulation, increasing its viscosity, which is consistent with previous reports. In the IRA complex, both HA and PN are high-MW polymers, making physical interactions and molecular entanglement possible. Additionally, they can interact indirectly through hydrogen bonding, particularly via their polar functional groups (e.g., the carboxyl (-COO^−^) groups of HA and the hydroxyl (-OH) groups of PN).

### 3.2. Antioxidant Capacity Assessments

**Figure 3:**
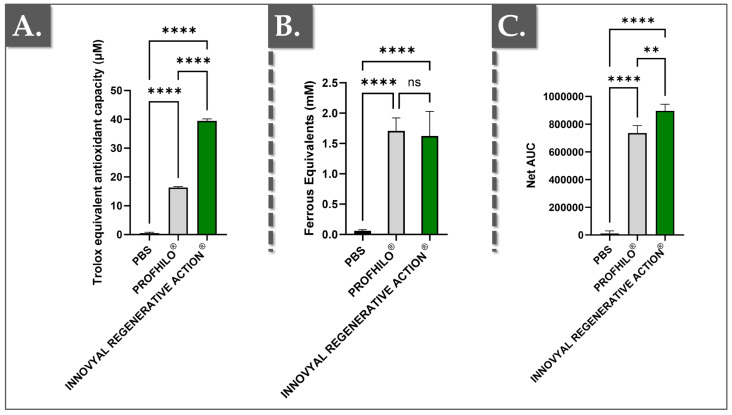
Comparative antioxidant capacities of IRA and Profhilo^®^ assessed by CUPRAC, FRAP, and ORAC assays. (**A**) Results of CUPRAC comparative antioxidant determination. (**B**) Results of FRAP comparative antioxidant determination. (**C**) Results of ORAC comparative antioxidant determination. A significance level described by two asterisks “**” corresponds to a *p*-value between 0.001 and 0.01. A significance level described by four asterisks “****” corresponds to a *p*-value below 0.0001. Statistical analysis details are presented in Table S5. AUC, area under the curve; ns, non-significant; PBS, phosphate-buffered saline.

In the skin, ROS are generated by exposure to ultraviolet (UV) radiation, pollution, and other environmental factors. These molecules can cause oxidative stress, leading to the degradation of collagen, elastin, and other critical components of the skin ECM, ultimately contributing to premature aging, inflammation, and skin diseases. Therefore, the antioxidant capacity of skincare formulations is crucial as it helps to neutralize ROS, preventing oxidative damage and maintaining skin health [44,45,46]. In order to study the products’ internal (i.e., finished product stability against oxidative degradation) and external (i.e., potential for in situ ROS scavenging) mechanisms of action, Figure 3 presents a comparative analysis of the antioxidant capacities of IRA and Profhilo^®^ using three different assays (FRAP, ORAC, and CUPRAC).

In the FRAP assay, antioxidants reduce Fe^3^⁺ to Fe^2^⁺, with ferrous equivalents quantified spectrophotometrically. The ORAC assay measures the protection against oxidative damage by tracking the fluorescence decay of fluorescein in the presence of radicals, with results expressed as the net area under the curve (AUC). The CUPRAC assay assesses the reduction of Cu^2^⁺ to Cu⁺ by antioxidants, with results expressed as Trolox equivalents [44,45]. All measurements were performed in triplicate, with the results normalized against phosphate-buffered saline (PBS).

The data presented in Figure 3 provide a comprehensive comparison of the antioxidant capacities of IRA and Profhilo^®^ across several assays, providing insights into their potential efficacy as skin boosters in aesthetic medicine. Importantly, IRA consistently demonstrated higher antioxidant capacities compared to Profhilo^®^ and the control (PBS). In the CUPRAC assay, IRA demonstrated significantly higher antioxidant capacity compared to Profhilo^®^ (i.e., more than 2 times), indicating superior efficacy in reducing copper ions, which are involved in oxidative stress pathways (Figure 3A). Of note, the CUPRAC assay is an excellent method for evaluating antioxidant capacity because it measures the electron-donating ability of antioxidants to reduce Cu^2^⁺ to Cu⁺. The redox potential plays a crucial role in the CUPRAC method as it dictates the energy required for the redox reaction. Cu^2^⁺, with a relatively low redox potential, facilitates faster reactions, making the CUPRAC method more selective for compounds that are less responsive to other assays [45,47].

Contrastingly, FRAP measures the immediate reducing power of antioxidants by evaluating their ability to reduce Fe^3^⁺ to Fe^2^⁺. The FRAP assay is well-known and considered a reliable and reproducible test [48]. IRA exhibited values slightly lower than Profhilo^®^, with no significant differences (Figure 3B). Nevertheless, both products indicated a stronger immediate reducing power than the PBS control.

Finally, the ORAC assay is particularly valuable for evaluating the antioxidant capacity of skin booster products because it measures the ability of antioxidants to protect against oxidative damage over time. The assay specifically targets peroxyl radicals, which are among the most reactive and damaging free radicals in the skin [49]. By monitoring the fluorescence decay of a probe over time, the ORAC assay provides a dynamic and comprehensive assessment of how well a product can sustain antioxidant protection. Therein, a ROS generator (i.e., AAPH [2,2′-azobis(2-methylpropionamidine) dihydrochloride]), which produces a peroxyl free radical (ROO•) upon thermal decomposition, is used in the ORAC assay.

Of note, AAPH is commonly used in vitro to induce oxidative stress and cellular senescence in skin cells or as an oxidation model in various studies [50,51]. Importantly, this free radical is commonly found in the body, making this reaction biologically relevant [50]. In the comparison between Profhilo^®^ and IRA, the higher ORAC values obtained for IRA indicated a superior ability to provide long-term protection against oxidative stress (Figure 3C). This sustained antioxidant activity is crucial in skincare, and aesthetic medicine in general, as it helps prevent the gradual degradation of skin components such as collagen and elastin, which are essential for maintaining skin firmness and elasticity. The strong comparative performance of IRA in the ORAC assay suggests that it is more effective in preventing the cumulative damage caused by prolonged exposure to environmental stressors (Figure 3C).

### 3.3. Bio-Stimulatory Attribute Assessments in a Skin Cell Model

**Figure 4:**
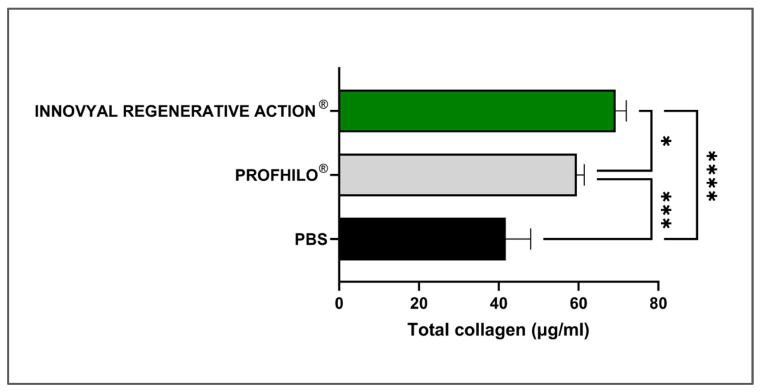
Absolute values of the total collagen produced by primary dermal fibroblasts incubated with the products for 72 h. Measurements were performed four times in triplicates and standard deviations were reported as error bars around mean values. A significance level described by one asterisk “*” corresponds to a *p*-value between 0.01 and 0.05. A significance level described by three asterisks “***” corresponds to a *p*-value between 0.0001 and 0.001. A significance level described by four asterisks “****” corresponds to a *p*-value below 0.0001. Statistical analysis details are presented in Table S6. PBS, phosphate-buffered saline.

Based on their clinical indications and the capacity of IRA and Profhilo^®^ to act as bio-stimulators, an in vitro dermal fibroblast model was established to relatively quantify total collagen induction. In aesthetic medicine, bio-stimulation is primarily characterized by the process of total collagen level stimulation [52,53]. Of note, it is often challenging to compare clinical case results between two different products due to the significant influence of injection techniques, differing mechanical properties of the gels, and inter-individual variability. In contrast, comparing collagen production by fibroblasts in a controlled experimental setup provides a straightforward approach to obtaining an initial assessment of a product’s bio-stimulatory capacity and its relative performance against comparators. The investigated products did not lower the viability of the cells following direct incubation in vitro (Figure S2). The total collagen production values by primary juvenile fibroblasts after 72 h of incubation with the test items are presented in Figure 4.

In the experimental setup, IRA and Profhilo^®^ were incubated in vitro in direct contact with human juvenile dermal fibroblasts for 72 h. The primary cells maintained their spindle-shaped phenotype throughout the assay and the hydrogels did not exhibit any cytotoxic effects. The cells were then detached and thermally lysed through cycles of freezing and thawing in order to measure the total collagen contents by fluorescence. The results highlighted a significant increase in collagen production by fibroblasts treated with IRA (Figure 4). Therein, a main formulation-based explanation is that IRA combines the well-known multimodal benefits of HA (i.e., which is the main component of Profhilo^®^) with the respective potent rejuvenation attributes of PN and niacinamide (Table 1).

Of note, PNs are high-MW DNA fragments (i.e., ≥1500 kDa), which have shown highly promising clinical efficacy in skin and hair rejuvenation by consensus of an Italian scientific board [19]. Specifically, PNs are clinically used in cutaneous rejuvenation, aiming for an increase in dermal fibroblast populations, increased collagen production in the skin ECM, and improved tissue circulation [18,19,20,21,22,23,24,25,26]. Additionally, an in vitro and in vivo murine study has demonstrated that PN-HA complex fillers can stimulate fibroblast proliferation, facilitate cutaneous volume increase, and promote skin regeneration [54]. Herein, IRA showed significantly higher collagen production in comparison with Profhilo^®^ and PBS. As mentioned, IRA also contains niacinamide, which is a key multi-functional ingredient in skincare and cosmetic products [55]. Indeed, numerous studies have shown that niacinamide stimulates fibroblasts to produce more collagen, supported by experimental designs both in vivo and ex vivo, particularly using skin explants isolated from patients undergoing abdominoplasty [56,57,58,59].

### 3.4. Clinical Case Reports on the Efficacy of IRA in Periocular Prejuvenation

**Figure 5:**
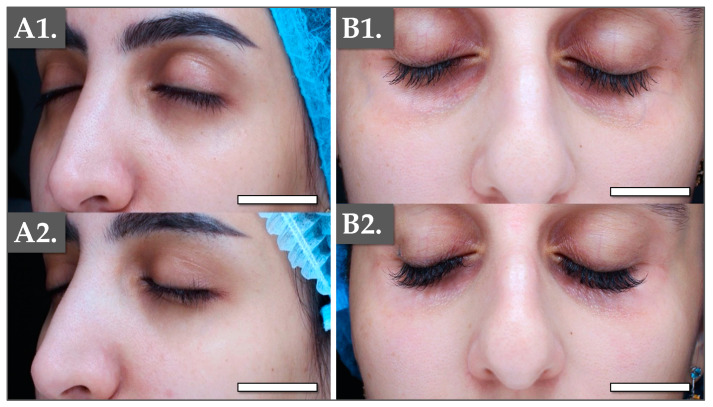
(**A1**,**A2**) Clinical imaging of the treatment sites for Patient N°1 before the IRA-based cosmetic intervention (**A1**) and at one month of clinical follow-up (**A2**). Scale bars = 25 mm. Clinical imaging of the treatment sites for Patient N°2 before the IRA-based cosmetic intervention (**B1**) and at two months of clinical follow-up (**B2**). Scale bars = 20 mm.

Based on the obtained in vitro datasets, the second part of the present study focused on the collection of pilot efficacy data for the use of IRA in periocular prejuvenation. Therefore, local monotherapy was carried out using IRA in two female patients. The product was injected intradermally (i.e., with a 30-gauge needle) using a multiple microaliquot technique. Specifically, volumes of 1.0 mL were applied to each side of the face. The treatment was administered twice with a two-week interval. Clinical visits for follow-up imaging were performed after one month or two months (Figure 5).

In detail, a 31-year-old medically healthy Saudi female patient (Patient N°1) consulted for fine periocular lines and bilateral discoloration, mainly on the lower eyelids. Upon clinical examination, no indication of cutaneous volume loss in the tear trough or in the palpebral-malar groove was evidenced (Figure 5(A1)). The patient expressed a preference against dermal fillers and sought a non-invasive solution for periocular appearance enhancement. In parallel, a 37-year-old Saudi female (Patient N°2), with no underlying medical conditions, consulted to address concerns about thin and transparent skin along with fine lines in the periocular area. As conventional fillers might have appeared too thick in the case of Patient N°2, IRA was administered using the same technique as described hereabove. Clinical follow-up imaging was performed two months post-treatment (Figure 5(A2,B2)).

In both clinical cases, adverse events were minimal and were limited to localized swelling, lasting for a maximum of five days, along with bruising at the injection sites. The latter was managed by selecting optimal procedure timing and by providing appropriate aftercare. Namely, an *Arnica montana* cream was locally applied as needed, supplemented by 20 mg of systemic Reparil^®^ three times per day for five days. Encouraging clinical effects of IRA in addressing periocular cutaneous early signs of aging were observed (i.e., improved texture and softened fine lines), and in its ability to perform tear trough correction (Figure 5). In the first case, a plumping effect was noticeable on the lower orbital area (i.e., the lower part of the under-eye hollow; Figure 5(A2)). A general brightening effect was also observed in both patients, along with smoothing out of fine lines. For Patient N°2, tissue restructuring was evident in the lateral orbital region, with a slight increase in tissue thickness (Figure 5(B2)). This change can alter light reflection, contributing to a reduction in discoloration and an overall improvement in appearance.

Of note, current market trends toward “tweakments” for skin prejuvenation align with the preferences of younger patients seeking non-invasive solutions to address early signs of aging. Importantly, successfully rejuvenated areas (i.e., the forehead, cheeks, and jawline) are known to draw attention to unrejuvenated areas, such as the periocular region. The IRA PN-HA regenerative complex administered in this study is marketed as a polyvalent hydrogel formulation, which combines the documented rejuvenation attributes of PN with the positive effects of HA and niacinamide in skincare [18]. The considered formulation yielded immediate and observable results, incurred short downtime, and holds the potential to simultaneously address multiple concerns for local appearance enhancement (Figure 5). Overall, IRA was preliminarily shown to offer a safe and effective monotherapy option for fine lines and skin discoloration improvement in the periocular region, meeting current clinician and patient demands for high-quality non-surgical cosmetic interventions.

### 3.5. Clinical Perspectives on the Functions of IRA

It was set forth that the described success of the IRA-based procedure, with minimal downtime, was due to the action of the PN-HA regenerative biopolymer complex (Figure 5). Importantly, since the periocular region perpetually evolves and is constituted by the thinnest skin in the human body, ad hoc dermal filler formulations should be reversible and liquid in behavior, contrasting with thick gel-like substances [12]. Therefore, linear HA fillers are usually clinically preferred. Notwithstanding, current trends fundamentally challenge the cosmetic market, with patients requiring less-invasive treatments and immediate results. Thus, clinical requirements are oriented toward formulations that simultaneously address multiple cutaneous concerns and mitigate aging signs. Therein, IRA combines the well-known multimodal benefits of HA with the respective potent rejuvenation attributes of PN and niacinamide (vitamin B3). Specifically, PNs are clinically used in cutaneous rejuvenation, aiming for an increase in dermal fibroblast populations, increased collagen production in the skin ECM, and improved tissue circulation. Additionally, PNs increase fibronectin contents, which are essential for wound healing, tissue regeneration, and for the maintenance of a balanced isometric skin hydration [25]. Thus, PN-based formulations are considered the most polyvalent skin boosters currently available on the market [19].

Importantly, comparative studies of PN-based fillers against non-cross-linked HA fillers for periocular rejuvenation have concluded that both are safe and effective [19]. Notably, both treatments improved skin elasticity, wherein PN fillers showed higher efficacy and provided long-lasting effects [20]. In a recent prospective clinical trial for correction of moderate-to-severe nasolabial folds, a highly purified PN-based product was used before cross-linked HA dermal filler administration, with PN significantly improving the dermal quality and texture at 3 and 6 months [17]. Notwithstanding, while several studies have demonstrated the effectiveness of HA- or PN-based fillers, less is known about the efficacy of monotherapy combined formulations (i.e., PN and non-cross-linked HA). Notably, an in vitro and in vivo murine study has demonstrated that PN-HA complex fillers can stimulate fibroblast proliferation, facilitate cutaneous volume increase, and promote skin regeneration [54]. Of key interest, IRA combines PN, niacinamide, and HA, where clinical monotherapy administration displayed fast and effective results for periocular prejuvenation and decreased visible hyperpigmentation (Figure 5). Furthermore, the original in vitro data have shown that such monotherapies may address dermal restructuring, notably via total collagen level increase promotion (Figure 4).

Of further note, IRA also contains niacinamide, a key multi-functional ingredient in skincare and cosmetic products. Specifically, niacinamide is a multi-purpose antiaging ingredient with robustly demonstrated antioxidative, anti-inflammatory, and anti-pigmentary activities. Thus, this vitamin is known to reverse some of the most important cutaneous structural changes associated with biological aging [54,59,60]. Overall, the action of niacinamide may be conjointly considered with that of PN and HA in order to scientifically support the reported clinical efficacy of IRA in managing periocular cutaneous discoloration and related early aging signs.

### 3.6. Study Limitations and Future Research Directions

Several limitations and methodological areas for improvement were identified for both the in vitro and in vivo portion of the presented study, along with perspectives for future translational research. Regarding the identified limitations of the present study, the reported pilot clinical results were limited to two case reports. Furthermore, quantitative measurement using instruments such as the Dermascan, Cutometer, Corneometer, as well as the Chromameter and Mexameter (i.e., for coloration change quantification) could be integrated in additional clinical work for an objective analysis of clinical parameters. A third technical limitation of the present study concerned the in vitro cell-based assays, which did not include orthogonal tests, notably for specific-collagen-type quantification (e.g., using proteomics, RT-PCR, and ELISA). Additionally, in vitro cell-based assays under different stimuli (e.g., under oxidative stress or UV) could be performed in order to better understand the reported mechanism of collagen induction. The fourth limitation was the number of retained comparative commercial products, which was due to the relatively small selection of formulas available for purchase.

Regarding future perspectives for this research, IRA could be the subject of a prospective clinical study over a period of 1 to 3 months to evaluate its effects on the skin using quantitative instrumental measurements for an enhanced objective analysis. An ex vivo study on cultured skin explants could also be conducted to assess the product in situ effects on collagen and elastin contents and to quantify the evolution of these two proteins of interest (i.e., as a proxy for skin quality).

## 4. Conclusions

The present study investigated a PN-HA regenerative biopolymer complex (Innovyal Regenerative Action^®^ [IRA]) for facial cutaneous prejuvenation. In vitro characterization demonstrated that IRA exhibited superior rheological properties compared to commercially available dermbooster products, suggesting its potential for better tissue remodeling stimulation and prolonged effect exertion. Furthermore, IRA demonstrated pronounced antioxidant capacities in various assays, indicating its ability to protect the skin from oxidative stress and premature aging. Notably, IRA significantly stimulated collagen production in human fibroblasts, confirming its bio-stimulatory potential. Finally, pilot clinical observations from two patients showed promising potential of IRA monotherapy in treating aging signs in the periocular region.

These preliminary findings suggested that IRA offers a promising approach for addressing the growing demand for minimally invasive, effective, and multi-functional prejuvenation treatments, particularly in the delicate periocular area. This study provides valuable preclinical and preliminary clinical observations supporting the use of the investigated PN-HA formulation, contributing to the advancement of prejuvenation techniques for addressing early signs of facial skin aging. The presented findings provide a strong foundation for future research, including prospective clinical trials and investigations into the long-term effects of the IRA formulation.

## Figures and Tables

**Table 1:** Technical overview of the regenerative HA-based aesthetic products that were retained for experimental rheological behavior quantification. NCTF, SCG, and Profhilo^®^ were included in this study as comparators based on product technical specification benchmarking. HA, hyaluronic acid; NA, non-applicable; PN, polynucleotide.

Product Commercial Name	HA Concentration ^1^	Total Biopolymer Concentration ^2^	Packaging	Main Composition ^3^	Manufacturing Technology
Innovyal Regenerative Action^®^ [IRA] or “HA-PN complex”	5 mg/mL	12.5 mg/mL	3 mL vial	HA, PN, vitamin B3	Boost & Shield^®^
Profhilo^®^	32 mg/mL	32 mg/mL	2 mL syringe	HA	NAHYCO^®^
Suisselle Cellbooster^®^ Glow [SCG]	6 mg/mL	6 mg/mL	3 mL vial	HA, 2 vitamins, and 6 amino acids	CHAC
NCTF^®^ 135 HA [NCTF]	5 mg/mL	5 mg/mL	5 mL vial	HA, 12 vitamins, 6 minerals, 5 nucleic acids, 24 amino acids, 6 coenzymes, glutathione, polysorbate 80, glucuronic acid, glucosamine, and dextrose	NA

^1^ Hyaluronic acid concentration in the product, as specified by the manufacturer. ^2^ Total biopolymer contents (i.e., addition of the biopolymer amounts, e.g., HA + PN concentrations). ^3^ Key ingredients listed by the manufacturer.

## Data Availability

The data presented in this study are openly available within the article files.

## Supplementary Materials

The following supporting information can be downloaded at https://www.mdpi.com/article/10.3390/polym17050638/s1, Figure S1: Complementary rheological data analysis; Figure S2: Cytotoxicity data; Table S1: Detailed statistical analyses for the data presented in Figure 1; Table S2: Numerical data for rheology analyses; Tables S3–S6: Detailed statistical analyses for the data presented in Figure 2, Figure 3 and Figure 4 and Figure S1.

## Abbreviations

AAPH	2,2′-azobis(2-amidinopropane) dihydrochloride
CUPRAC	Cupric reducing antioxidant capacity
DMEM	Dulbecco’s modified Eagle medium
DNA	Deoxyribonucleic acid
ECM	Extracellular matrix
ELISA	Enzyme-linked immunosorbent assay
FBS	Fetal bovine serum
FRAP	Ferrous reduction antioxidant power
G′	Storage modulus
G″	Loss modulus
HA	Hyaluronic acid
IRA	Innovyal Regenerative Action product
min	Minute
MW	Molecular weight
NA	Non-applicable
NCTF	NCTF 135 HA product
ns	Non-significant
ORAC	Oxygen radical antioxidant capacity
Pa	Pascals
Pa·s	Pascal seconds
PBS	Phosphate-buffered saline
PDRN	Polydeoxyribonucleotide
PN	Polynucleotide
RFMN	Radiofrequency microneedling
ROS	Reactive oxygen species
s	Second
SCG	Suisselle Cellbooster Glow product
USA	United States of America
UV	Ultraviolet
VEGF	Vascular endothelial growth factor
