# Supplementary material for: In Vitro Evaluation and Clinical Effects of a Regenerative Complex with Non-Cross-Linked Hyaluronic Acid and a High-Molecular-Weight Polynucleotide for Periorbital Treatment
Source: Polymers (Basel). 2025 Feb 27;17(5):638. doi: 10.3390/polym17050638 (PMC11902836; doi:10.3390/polym17050638)
Supplement: Supplementary file 1 [file polymers-17-00638-s001.zip › polymers-3435671-supplementary.pdf]

polymers

MDPI

Supplementary Materials:

In Vitro Evaluation and Clinical Effects of a Regenerative Complex with Non-Cross-Linked Hyaluronic Acid and a High-Molecular-Weight Polynucleotide for Periorbital Treatment

Supplementary Figures

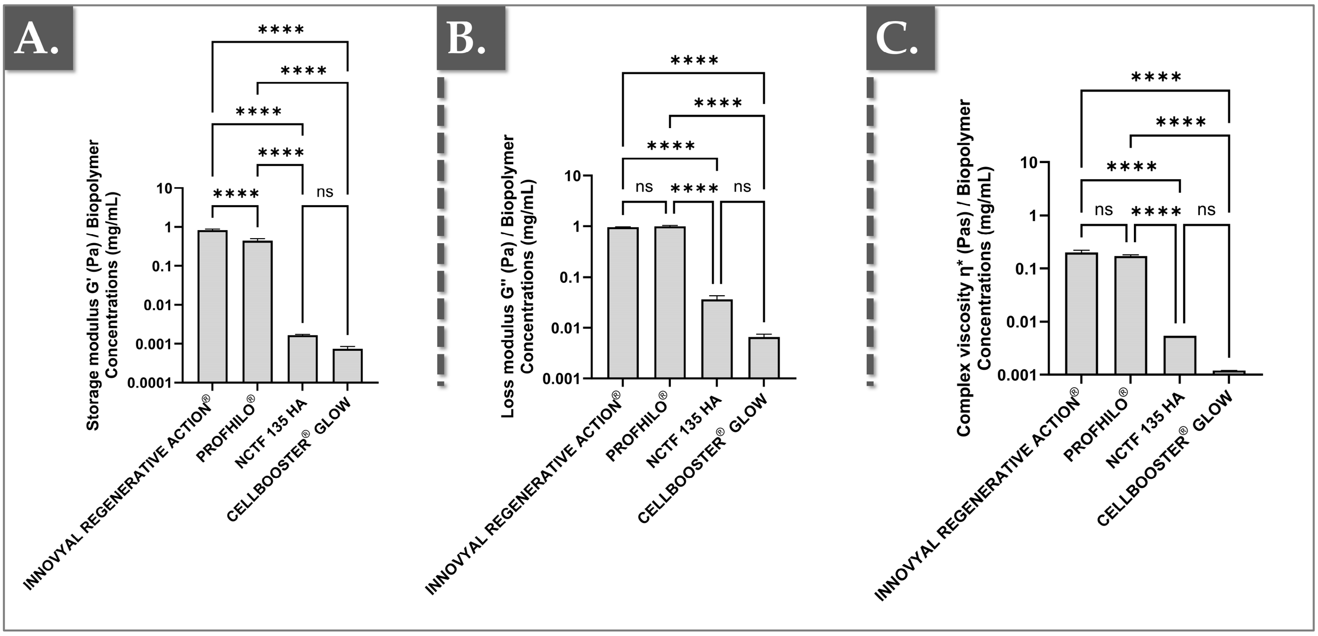

Figure S1. Rheological data complementing the results presented in Figure 2. The rheological values were normalized to the total biopolymer contents of the products. (**A**) Normalized storage moduli (G′) comparison for the investigated products. (**B**) Normalized loss moduli (G′′) comparison for the investigated products. (**C**) Normalized complex viscosity (η*) comparison for the investigated products. A significance level described by four asterisks "****" corresponds to a p-value below 0.0001. Statistical analysis details are presented in Table S4. Ns, non-significant; Pa, Pascals; Pa·s, Pascal seconds.

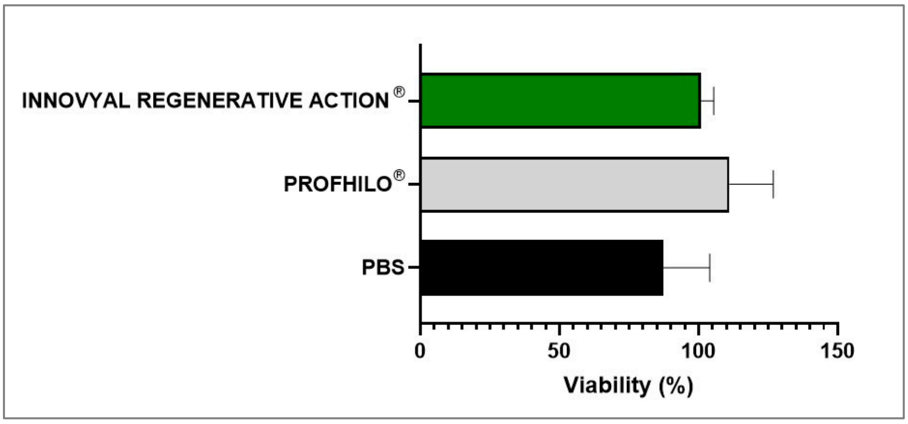

 https://doi.org/10.3390/polym17050638

Figure S2. Cytotoxicity data complementing the results presented in Figure 4, obtained after 72 h of treatment with the samples. The investigated products did not lower the viability of the cells following direct incubation in vitro. PBS, phosphate-buffered saline.

2. Supplementary Tables

Table S1. Quantitative results of Tukey's post hoc multiple comparison test in relation to the comparative rheological data presented in Figure 1. Non-significant differences correspond to a *p*-value > 0.05. ns, non-significant; Pa, Pascals.

Parameter	Compared Groups	Mean Absolute Difference	Adjusted *p*-Value	Significance Level [1]
Storage Modulus G′ (Pa)	INNOVYAL REGENERATIVE ACTION® vs. NCTF 135 HA	10.34	< 0.0001	****
	INNOVYAL REGENERATIVE ACTION® vs. SUISSELLE CELLBOOSTER® GLOW	10.35	< 0.0001	****
	INNOVYAL REGENERATIVE ACTION® vs. PROFHILO®	−3.82	0.0054	**
	NCTF 135 HA vs. SUISSELLE CELLBOOSTER® GLOW	0.004	> 0.9999	ns
	NCTF 135 HA vs. PROFHILO®	−14.16	< 0.0001	****
	SUISSELLE CELLBOOSTER® GLOW vs. PROFHILO®	−14.17	< 0.0001	****
Loss Modulus G′′ (Pa)	INNOVYAL REGENERATIVE ACTION® vs. NCTF 135 HA	11.82	< 0.0001	****
	INNOVYAL REGENERATIVE ACTION® vs. SUISSELLE CELLBOOSTER® GLOW	11.96	< 0.0001	****
	INNOVYAL REGENERATIVE ACTION® vs. PROFHILO®	−19.78	< 0.0001	****
	NCTF 135 HA vs. SUISSELLE CELLBOOSTER® GLOW	0.1415	0.9944	ns
	NCTF 135 HA vs. PROFHILO®	−31.6	< 0.0001	****
	SUISSELLE CELLBOOSTER® GLOW vs. PROFHILO®	−31.74	< 0.0001	****
Complex Viscosity η*	INNOVYAL REGENERATIVE ACTION® vs. NCTF 135 HA	2.48	< 0.0001	****
	INNOVYAL REGENERATIVE ACTION® vs. SUISSELLE CELLBOOSTER® GLOW	2.50	< 0.0001	****
	INNOVYAL REGENERATIVE ACTION® vs. PROFHILO®	−2.99	< 0.0001	****
	NCTF 135 HA vs. SUISSELLE CELLBOOSTER® GLOW	0.02	0.9994	ns
	NCTF 135 HA vs. PROFHILO®	−5.47	< 0.0001	****
	SUISSELLE CELLBOOSTER® GLOW vs. PROFHILO®	−5.489	< 0.0001	****
Tan δ	INNOVYAL REGENERATIVE ACTION® vs. NCTF 135 HA	−20.39	< 0.0001	****
	INNOVYAL REGENERATIVE ACTION® vs. SUISSELLE CELLBOOSTER® GLOW	−7.73	0.0014	**
	INNOVYAL REGENERATIVE ACTION® vs. PROFHILO®	−1.11	0.8232	ns
	NCTF 135 HA vs. SUISSELLE CELLBOOSTER® GLOW	12.66	< 0.0001	****
	NCTF 135 HA vs. PROFHILO®	19.28	< 0.0001	****
	SUISSELLE CELLBOOSTER® GLOW vs. PROFHILO®	6.621	0.0038	**

[1] A significance level described by two asterisks "**" corresponds to a *p*-value between 0.001 and 0.01. A significance level described by four asterisks "****" corresponds to a *p*-value below 0.0001.

Table S2. Numerical values in relation to the comparative rheological data presented in Figure 1 and Figure 2. Pa, Pascals.

Product	G' (Pa)	G'' (Pa)	η* (Pa·s)	G'/[HA]	G''/[HA]	η*/[HA]
INNOVYAL REGENERATIVE ACTION®	10.35 ± 0.721	12.00 ± 0.175	2.51 ± 0.26	2.07 ± 0.144	2.400 ± 0.035	0.201 ± 0.021
NCTF 135 HA	0.008 ± 0.001	0.181 ± 0.031	0.027 ± 0.001	0.002 ± 0.000	0.0362 ± 0.006	0.005 ± 0.000
SUISELLE CELLBOOSTER® GLOW	0.005 ± 0.001	0.040 ± 0.005	0.007 ± 0.001	0.001 ± 0.000	0.00658 ± 0.001	0.001 ± 0.000
PROFHILO®	14.170 ± 1.779	31.777 ± 1.403	5.497 ± 0.319	0.443 ± 0.056	0.993 ± 0.044	0.172 ± 0.010

Table S3. Quantitative results of Tukey's post hoc multiple comparison test in relation to the comparative rheological data presented in Figure 2. Non-significant differences correspond to a *p*-value > 0.05. ns, non-significant; Pa, Pascals.

Parameter	Compared Groups	Mean Absolute Difference	Adjusted *p*-Value	Significance Level [1]
Storage Modulus G′ (Pa)	INNOVYAL REGENERATIVE ACTION® vs. NCTF 135 HA	2.068	< 0.0001	****
	INNOVYAL REGENERATIVE ACTION® vs. SUISSELLE CELLBOOSTER® GLOW	2.069	< 0.0001	****
	INNOVYAL REGENERATIVE ACTION® vs. PROFHILO®	1.627	< 0.0001	****
	NCTF 135 HA vs. SUISSELLE CELLBOOSTER® GLOW	0.001	> 0.9999	ns
	NCTF 135 HA vs. PROFHILO®	–0.441	0.0005	***
	SUISSELLE CELLBOOSTER® GLOW vs. PROFHILO®	–0.442	0.0005	***
Loss Modulus G′′ (Pa)	INNOVYAL REGENERATIVE ACTION® vs. NCTF 135 HA	2.364	< 0.0001	****
	INNOVYAL REGENERATIVE ACTION® vs. SUISSELLE CELLBOOSTER® GLOW	2.393	< 0.0001	****
	INNOVYAL REGENERATIVE ACTION® vs. PROFHILO®	1.407	< 0.0001	****
	NCTF 135 HA vs. SUISSELLE CELLBOOSTER® GLOW	0.030	0.5965	ns
	NCTF 135 HA vs. PROFHILO®	–0.957	< 0.0001	****
	SUISSELLE CELLBOOSTER® GLOW vs. PROFHILO®	–0.987	< 0.0001	****
Complex Viscosity η*	INNOVYAL REGENERATIVE ACTION® vs. NCTF 135 HA	0.497	< 0.0001	****
	INNOVYAL REGENERATIVE ACTION® vs. SUISSELLE CELLBOOSTER® GLOW	0.501	< 0.0001	****
	INNOVYAL REGENERATIVE ACTION® vs. PROFHILO®	0.330	< 0.0001	****
	NCTF 135 HA vs. SUISSELLE CELLBOOSTER® GLOW	0.004	0.9971	ns
	NCTF 135 HA vs. PROFHILO®	–0.166	0.0003	***
	SUISSELLE CELLBOOSTER® GLOW vs. PROFHILO®	–0.171	0.0002	***

[1] A significance level described by three asterisks "***" corresponds to a *p*-value between 0.0001 and 0.001. A significance level described by four asterisks "****" corresponds to a *p*-value below 0.0001.

Table S4. Quantitative results of Tukey's post hoc multiple comparison test in relation to the comparative rheological data presented in Figure S1. Non-significant differences correspond to a *p*-value > 0.05. ns, non-significant; Pa, Pascals.

Parameter	Compared Groups	Mean Absolute Difference	Adjusted *p*-Value	Significance Level [1]
Storage Modulus G′ (Pa)	INNOVYAL REGENERATIVE ACTION® vs. NCTF 135 HA	0.8263	< 0.0001	****
	INNOVYAL REGENERATIVE ACTION® vs. SUISSELLE CELLBOOSTER® GLOW	0.8273	< 0.0001	****
	INNOVYAL REGENERATIVE ACTION® vs. PROFHILO®	0.3852	< 0.0001	****
	NCTF 135 HA vs. SUISSELLE CELLBOOSTER® GLOW	0.0009	> 0.9999	ns
	NCTF 135 HA vs. PROFHILO®	–0.4411	< 0.0001	****
	SUISSELLE CELLBOOSTER® GLOW vs. PROFHILO®	–0.4421	< 0.0001	****
Loss Modulus G′′ (Pa)	INNOVYAL REGENERATIVE ACTION® vs. NCTF 135 HA	0.9238	< 0.0001	****
	INNOVYAL REGENERATIVE ACTION® vs. SUISSELLE CELLBOOSTER® GLOW	0.9534	< 0.0001	****
	INNOVYAL REGENERATIVE ACTION® vs. PROFHILO®	–0.03305	0.3645	ns
	NCTF 135 HA vs. SUISSELLE CELLBOOSTER® GLOW	0.02962	0.4491	ns
	NCTF 135 HA vs. PROFHILO®	–0.9568	< 0.0001	****
	SUISSELLE CELLBOOSTER® GLOW vs. PROFHILO®	–0.9865	< 0.0001	****
Complex Viscosity ɳ*	INNOVYAL REGENERATIVE ACTION® vs. NCTF 135 HA	0.1954	< 0.0001	****
	INNOVYAL REGENERATIVE ACTION® vs. SUISSELLE CELLBOOSTER® GLOW	0.1996	< 0.0001	****
	INNOVYAL REGENERATIVE ACTION® vs. PROFHILO®	0.02903	0.0592	ns
	NCTF 135 HA vs. SUISSELLE CELLBOOSTER® GLOW	0.00422	0.9681	ns
	NCTF 135 HA vs. PROFHILO®	–0.1663	< 0.0001	****
	SUISSELLE CELLBOOSTER® GLOW vs. PROFHILO®	–0.1706	< 0.0001	****

[1] A significance level described by four asterisks "****" correspond to a *p*-value below 0.0001.

Table S5. Quantitative results of Tukey's post hoc multiple comparison test in relation to the comparative antioxidant activity data presented in Figure 3. Non-significant differences correspond to a *p*-value > 0.05. ns, non-significant; Pa, Pascals.

Parameter	Compared Groups	Mean Absolute Difference	Adjusted *p*-Value	Significance Level [1]
**CUPRAC**	PBS vs. INNOVYAL REGENERATIVE ACTION®	–38.96	< 0.0001	****
	PBS vs. PROFHILO®	–15.83	< 0.0001	****
	INNOVYAL REGENERATIVE ACTION® vs. PROFHILO®	23.13	< 0.0001	****
**FRAP**	PBS vs. INNOVYAL REGENERATIVE ACTION®	–1.563	< 0.0001	****
	PBS vs. PROFHILO®	–1.648	< 0.0001	****
	INNOVYAL REGENERATIVE ACTION® vs. PROFHILO®	–0.086	0.9007	ns
**ORAC**	PBS vs. INNOVYAL REGENERATIVE ACTION®	–885575	< 0.0001	****
	PBS vs. PROFHILO®	–725641	< 0.0001	****

	INNOVYAL REGENERATIVE ACTION® vs. PROFHILO®	159934	0.0090	**

[1] A significance level described by two asterisks "**" corresponds to a *p*-value between 0.001 and 0.01. A significance level described by four asterisks "****" corresponds to a *p*-value below 0.0001.

Table S6. Quantitative results of Tukey's post hoc multiple comparison test in relation to the comparative neocollagenesis stimulation data presented in Figure 4. Non-significant differences correspond to a *p*-value > 0.05. ns, non-significant; Pa, Pascals.

Parameter	Compared Groups	Mean Absolute Difference	Adjusted *p*-Value	Significance Level [1]
Collagen	PBS vs. INNOVYAL REGENERATIVE ACTION®	–27.560	< 0.0001	****
	PBS vs. PROFHILO®	–17.840	0.0004	***
	INNOVYAL REGENERATIVE ACTION® vs. PROFHILO®	9.727	0.0203	*

[1] A significance level described by one asterisk "*" corresponds to a *p*-value between 0.01 and 0.05. A significance level described by three asterisks "***" corresponds to a *p*-value between 0.0001 and 0.001. A significance level described by four asterisks "****" corresponds to a *p*-value below 0.0001.
